# An ameliorative protocol for the quantification of purine 5′,8-cyclo-2′-deoxynucleosides in oxidized DNA

**DOI:** 10.3389/fchem.2015.00047

**Published:** 2015-07-28

**Authors:** Michael A. Terzidis, Chryssostomos Chatgilialoglu

**Affiliations:** ^1^Istituto per la Sintesi Organica e la Fotoreattività, Consiglio Nazionale delle RicercheBologna, Italy; ^2^Institute of Nanoscience and Nanotechnology, National Centre of Scientific Research “Demokritos”Athens, Greece

**Keywords:** DNA damage, cyclonucleosides, enzymatic digestion protocols, isotope dilution analysis, liquid chromatography-tandem mass spectrometry

## Abstract

5′,8-Cyclo-2′-deoxyadenosine (cdA) and 5′,8-cyclo-2′-deoxyguanosine (cdG) are lesions resulting from hydroxyl radical (HO^**·**^) attack on the 5′H of the nucleoside sugar moiety and exist in both 5′*R* and 5′*S* diastereomeric forms. Increased levels of cdA and cdG are linked to Nucleotide Excision Repair (NER) mechanism deficiency and mutagenesis. Discrepancies in the damage measurements reported over recent years indicated the weakness of the actual protocols, in particular for ensuring the quantitative release of these lesions from the DNA sample and the appropriate method for their analysis. Herein we report the detailed revision leading to a cost-effective and efficient protocol for the DNA damage measurement, consisting of the nuclease benzonase and nuclease P1 enzymatic combination for DNA digestion followed by liquid chromatography isotope dilution tandem mass spectrometry analysis.

## Introduction

**Figure 1:**
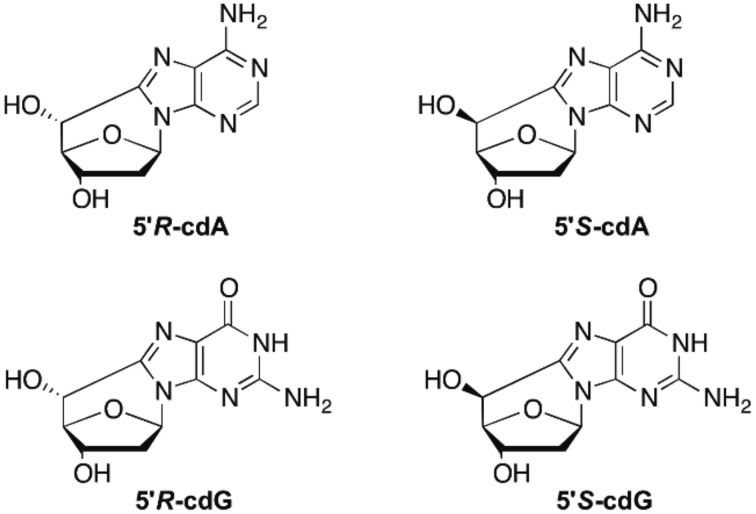
**5′,8-Cyclo-2′-deoxyadenosine (cdA) and 5′,8-cyclo-2′-deoxyguanosine (cdG) lesions**.

Hydroxyl radicals (HO^**·**^) are known for their reactivity toward DNA leading to nucleobase modifications and strand breaks. In particular, the attack at H5′ of DNA by HO^**·**^ is estimated to occur with a 55% probability over all possible sugar positions, and produces the C5′ radical (Aydogan et al., 2002). The chemistry of C5′ radical is very peculiar with respect to the other positions of 2-deoxyribose in the sense that it does not generate an abasic site, but unique cyclic base-sugar adducts are formed denominated cyclopurine lesions (Figure 1). Therefore, apart from the usual glycosidic bond, another covalent bond between the C5′ sugar and the C8 purine carbon atoms is present (Chatgilialoglu et al., 2011). 5′,8-Cyclo-2′-deoxyadenosine (cdA) and 5′,8-cyclo-2′-deoxyguanosine (cdG) in their 5′*R* and 5′*S* diastereomeric forms are tandem-type lesions observed among the DNA modifications and identified in mammalian cellular DNA *in vivo* (Chatgilialoglu et al., 2011). Nowadays these lesions are considered as robust biomarkers for oxidative stress and might be resistant to repair machinery in cells. Indeed, it has been recently found that these tandem lesions accumulate with aging in a tissue-specific manner (liver > kidney > brain), providing evidence that DNA repair mechanisms are inadequate to preserve the integrity of genetic material from these lesions (Wang et al., 2011, 2012). Results from competitive transcription and adduct bypass assay revealed that 5′*S*-cdG strongly inhibits transcription *in vitro* and in mammalian cells, and induces transcriptional mutagenesis both *in vitro* and *in vivo* (You et al., 2012). Recent studies reported cyclopurine lesions as reliable oxidative stress biomarkers in animal models for examination of liver injury pathophysiology in Wilson's disease and pigmentation (Wang et al., 2011; Mitra et al., 2012). A direct comparison of relative Nucleotide Excision Repair (NER) efficiencies of the four cyclopurines has been determined in the identical sequence contexts and in the same HeLa cell extract preparations (Kropachev et al., 2014). The cdA and cdG lesions were excised with similar efficiencies, but the efficiencies for both 5′*R* cyclopurines were greater by a factor of ~2 than for the 5′*S* lesions. Based on molecular modeling and molecular dynamics simulations, the structural and energetic origins of this difference in NER efficiencies have been suggested. Indeed, the greater stacking impairment in the 5′*R* stereoisomer correlates well with its greater relative NER excision efficiency.

Modified oligonucleotides and dimers containing the cyclopurines were synthesized and used by different research groups for the development of enzymatic protocols aiming at the quantitative liberation of the lesions as nucleoside units, prior to quantification (Romieu et al., 1998, 1999; Jaruga et al., 2004). The cocktails used in the latter studies contain the nuclease enzyme P1, which is dependent on both zinc and water co-factors and is known to recognize and hydrolyze single-stranded DNA and RNA to the level of 5′-mononucleotides by concerted endo- and exonucleolytic action (Fujimoto et al., 1975; Volbeda et al., 1991; Romier et al., 1998). The mechanism behind the action of the snake venom phosphodiesterase (3′-exo) and phosphodiesterase II (5′-exo), also used in digestion mixtures, has been identified to proceed through the formation of a nucleotidyl-enzyme intermediate by an active-site threonine insult, on which a double-displacement takes place (Culp et al., 1985; Cummins and Potter, 1987). Results reported for irradiated samples of calf thymus DNA have been critically reviewed, highlighting that the available protocols are not the best performing test available, and revision is needed in order to get precise damage values and foster the potential involvement of these lesions in human health (Chatgilialoglu et al., 2011). The objectives of this work were to (i) develop and validate a sensitive analytical method based on the stable isotope-dilution tandem mass spectrometry technique using triple quadrupole mass spectrometry, and (ii) to elucidate the hydrolytic action of several enzymes for the satisfactory DNA digestion and quantitative release of single nucleoside cyclopurines. We anticipate that our revised protocol, consisting of the nuclease benzonase and nuclease P1 enzymatic combination for DNA digestion followed by liquid chromatography isotope dilution tandem mass spectrometry analysis, provides benefits for costs and efficiency related to DNA damage measurement including cyclopurine lesions.

## Materials and methods

### Chemicals

8-Bromo-2′-deoxyadenosine, 2′-deoxyadenosine monohydrated, 7,8-dihydro-8-oxo-2′-deoxyadenosine (8-oxo-dA), 2′-deoxyguanosine monohydrated, 7,8-dihydro-8-oxo-2′-deoxyguanosine 8-oxo-dG), and 8-bromo-2′-deoxyguanosine were purchased from Berry & Associates Inc. (Dexter, USA). [^15^*N*_5_]-2′-deoxyadenosine monohydrated and [^15^*N*_5_]-2′-deoxyguanosine monohydrated (all >98% isotopic purity) were purchased from Cambridge Isotope Laboratories (Andover, USA). All the salts and solvents, thymidine, 2′-deoxycytidine, activated calf thymus DNA, nuclease P1 from *Penicillium citrinum*, phosphodiesterase II, phosphodiesterase I from *Crotalus Adamanteus venom*, DNAse I, DNAse II, alkaline phosphatase from bovine intestinal mucosa, erythro-9-(2-hydroxy-3-nonyl)adenine hydrochoride (EHNA), benzonase 99%, deferoxamine mesylate salt, BHT and pentostatin, were obtained from Sigma (Taufkirchen, Germany and Milan, Italy) while the 3 kDa filters were purchased from Millipore (Bedford, USA). Distilled and deionized water (ddH2O) was purified by a Milli-Q system (Millipore, Bedford, USA).

### Synthesis of reference compounds and internal standards

The four diastereoisomers 5′*R*-cdA, 5′*S*-cdA, 5′*R*-, and 5′*S*-cdG were synthesized according to published synthetic protocol (Jimenez et al., 2004; Terzidis and Chatgilialoglu, 2013) (e.g., see Figure S1 for cdA). Synthesis of ^15^*N* labeled of adenine derivatives was performed as previously reported for the unlabeled compounds (Boussicault et al., 2008). In particular, 1 mL of aqueous solution containing 1.5 mM of [^15^*N*_5_]-dA was prepared, flushed with N_2_O for 15 min and γ-irradiated with a total dose of 2 kGy at a dose rate of 4.5 Gy min^−1^. After the reaction crude mixture was submitted to HPLC purification and the peaks corresponding to [^15^*N*_5_]-5′*R*-cdA, [^15^*N*_5_]-5′*S*-cdA, and [^15^*N*_5_]-8-oxo-dA were collected (Figures S2, S3). The two diastereoisomers in ESI MS/MS analysis gave m/z 255 for the [M+H]^+^ and the two diagnostic fragment ions at m/z 169 and at m/z 141, whereas [^15^*N*_5_]-8-oxo-dA gave m/z 273 for the [M + H]^+^ and two fragment ions at m/z 157 and m/z 130, which correspond to the protonated 2-deoxyribose and [^15^*N*_5_]-8-oxo-adenine, respectively. The synthesis of [^15^*N*_5_]-8-bromo-2′-deoxyguanosine has been achieved starting from [^15^*N*_5_]-dG and following a known procedure for the unlabeled compound (Münzel et al., 2011). In particular, 1 mg (3.5 μmol) of [^15^*N*_5_]-dG was suspended to 500 μL of acetonitrile/water mixture 4:1 in a microcentrifuge tube of 1.5 mL volume. Then 1.3 mg *N*-bromosuccinimide (7.3 μmol) was added to the suspension in three portions and the mixture stirred at room temperature for 2 h. After the solvent was removed under a stream of argon, acetone (100 μL) was added. The mixture was stirred at room temperature for 4 h and then stored at −20°C overnight. Next, the mixture was centrifuged at 1000 g for 1 min and the supernatant was removed with a pipette. The precipitate was washed with cold acetone (60 μL), centrifuged again and the liquid removed with a pipette. The bromo derivative was irradiated as previously reported for the unlabeled compounds (Chatgilialoglu et al., 2007): 1 mM solution in ddH_2_O was transferred into the UV irradiation apparatus, flushed with argon for 15 min, and irradiated for 30 min with a UV light (5 W low pressure Hg lamp). The reaction mixture was quenched with a 5% NaHCO_3_ solution (final pH 7) and submitted to HPLC for purification (Figure S4) affording the two diastereomers [^15^*N*_5_]-5′*R*-cdG and [^15^*N*_5_]-5′*S*-cdG (Figures S4, S5). [^15^*N*_5_]-8-oxo-dG was prepared following a literature protocol (Taghizadeh et al., 2008). In particular, [^15^*N*_5_]-dG (0.24 mg, 0.82 μmol) was dissolved in 164 μL of water in an ultrasound bath. After, 5.45 μL of 0.5 M freshly prepared ascorbic acid (2.7 μmol) were added followed by 3.3 μL of 0.1 M CuSO_4_ (3.3 μmol) and 9.4 μL of 30% hydrogen peroxide. The reaction mixture was stirred at room temperature and quenched after 2 h with a 5% Na_2_SO_3_ solution. The crude was purified by HPLC (Figures S6, S7). The two diastereoisomers in ESI MS/MS analysis gave m/z 271 for the [M + H]^+^ and the two diagnostic fragment ions at m/z 185 and at m/z 206, whereas [^15^*N*_5_]-8-oxo-dG gave m/z 289 for the [M + H]^+^ and two fragment ions at m/z 173 and m/z 145, which correspond to the protonated 2-deoxyribose and [^15^*N*_5_]-8-oxo-guanine respectively. The isotopic purities of the ^15^*N* labeled compounds were found to be >99.9% by tandem mass spectrometry analysis using an ion-trap Bruker Daltonics MS^n^ system. The calculations were based on the ratio between the signals of the fragment ions corresponding to the labeled and the natural compounds. Their UV spectra were identical to those of the unlabeled compounds.

### Determination of purine 5′,8-cyclo-2′-deoxynucleosides extinction coefficients

The purity of the natural standard references of purine 5′,8-cyclo-2′-deoxynucleosides was determined after the synthesis according to published procedures (Jimenez et al., 2004; Terzidis and Chatgilialoglu, 2013). They were all controlled by HPLC-UV and LC-MS^2^ (Agilent-Bruker Daltonics ion trap) for purity. The purine 5′,8-cyclo-2′-deoxynucleosides obtained by the previous procedure were desalinated using solid phase extraction cartridges (Waters Sep-Pak® C18-E, 120 Å, 360 mg) by following the manufacturer's instruction. For the determination of the extinction coefficients (ε) at the λ_max_ of each nucleoside and, in order to reduce pipetting errors, aqueous solutions of each were prepared involving a five digital balance. The UV spectra were recorded by a UV spectrophotometer.

### Isotope dilution tandem mass spectrometry quantification

The quantification of the lesions was carried out by liquid chromatography isotope dilution tandem mass spectrometry technique. The calibration of the spectrometer with the natural lesions was based on the parameters reported previously (Belmadoui et al., 2010). The analytes were resolved on a 2 mm × 150 mm Luna C18 (2) 100 Å column (3 μ min particle size, Phenomenex) loaded with a pre-column C18 (2) cartridge using an LC system (Perkin Elmer Inc., USA) coupled with an AB Sciex 4000 triple quadrupole mass spectrometer (AB Sciex Inc., USA) working on multiple reaction monitoring mode. The chromatographic method used for the separation of the analytes started with 99% of 2 mM ammonium formate (solvent A) and 1% acetonitrile (solvent B). A gradient program using 2 mM ammonium formate (A) and acetonitrile (B) was involved, 0 → 20 min solvent B 1 → 9.8%. The system was washed for extra 5 min with isocratic solution B 15% and additional 10 min were given for re-equilibration after each analysis. The flow rate remained constant at 0.2 mL/min, the column was thermostated at 30°C and the injection volume was 15 μL. Linear responses were found for injection volumes up to 30 μL and upon dilution (see Figures S8, S10, respectively, in Supplementary Material).

### Preparation of the calibration curves

The construction of the calibration curves of the isotopic ^15^*N*_5_ labeled and unlabeled lesions of the four purine 5′,8-cyclo-2′-deoxynucleosides and the two 8-oxo-7,8-dihydro-2′-deoxynucleosides were prepared by plotting the MRM signal area ratios of the lesions and their isotopic equivalents against their corresponding concentration of the lesions. The concentration of the labeled lesions were chosen carefully be close to the concentration of the analytes in the samples (nM) and kept constant both for the construction of the curves and the quantification of the lesions in the samples. The latter was based on the comparison of the MRM signal areas between the lesions and their internal standards, and their respective response curves (see Table S2 in Supplementary Material). For this reason the horizontal axis values in the curves are given as analyte concentration (Figure S9). The concentrations of the lesions used for building up the calibration curves are reported in Table S3. For the preparation of the different solutions a five-digital balance was used in order to avoid pipetting errors. The determination of concentrations of the internal standard solutions containing the purine [^15^*N*_5_]-5′,8-cyclo-2′-deoxynucleosides and [^15^*N*_5_]-7,8-dihydro-8-oxo-2′-deoxyadenosine was based on their traces after HPLC-UV analyses (at 260 nm) and the corresponding response curves of the unlabeled equivalent compounds. The quantification of the [^15^*N*_5_]-7,8-dihydro-8-oxo-2′-deoxyguanosine solution was performed by UV spectroscopy.

### HPLC-UV analysis, off-line sample cleanup and enrichment

HPLC-UV analysis, cleanup and enrichment of the samples were performed on a 4.6 mm × 150 mm Luna C18 (2) 100 Å column (5 μ min particle size, Phenomenex) loaded with a pre-column C18 (2) cartridge, on an Agilent 1100 HPLC-UV system (Agilent, USA). The gradient program used an eluent composed by 2 mM ammonium formate (solvent A), acetonitrile (solvent B), and methanol (solvent C) (Table S5). The time windows used for the collection of fractions containing the lesions are reported in Table S6 (Figure S11). The collected fractions were freeze-dried, pooled, freeze-dried again, and redissolved in 50 μL ddH_2_O before LC-MS/MS analysis. The quantification of the dA, dG, dC, and Thy was based on their absorbance at 260 nm (Cui et al., 2013). The same analytical protocol was used also for the quantification of the normal 2′-deoxyribonucleosides in the enzymatic digestion studies.

### Lesion stability under acidic conditions

The stability of the lesions against the acidic hydrolysis was also investigated. Six aqueous mixtures of 10 μL containing a mixture of 19 pmol 8-oxo-dG, 6 pmol 5′*R*-cdG, 1 pmol 5′*S*-cdG, 5 pmol 8-oxo-dA, 5 pmol 5′*R*-cdA and 3 pmol 5′*S*-cdA were prepared and 10 μL of formic acid solutions with different concentrations were added in order to reach 0, 0.1, 0.5, 1, 5, and 10% final formic acid concentrations. The solutions were incubated at 37°C for 4 h and then quenched with cold NH_4_OH solution (Das et al., 2012). The mixture of internal standard references (labeled) was added. The samples were freeze-dried, reconstituted in 50 μL of ddH_2_O and analyzed by LC-MS/MS. The experiments were repeated three times.

### Enzymatic digestion protocols

Protocol A (Wang et al., 2011): 40 μg DNA were dissolved in 10 μL of Ar flushed buffer containing 0.3 M AcONa pH 5.6, 10 mM ZnCl_2_, 3 mM deferoxamine mesylate, and 1 mM EHNA. Next, 4 U of Nuclease P1 (in 30 mM AcONa pH 5.3, 5 mM ZnCl_2_ and 50 mM NaCl) and 5 mU phosphodiesterase II were added and the mixture was incubated in 37°C. After 48 h, 20 μL of 0.5 M Tris-HCl buffer pH 8.9 flushed with Ar were added along with 4 U of alkaline phosphatase and 5 mU of phosphodiesterase I. The mixture was incubated for extra 2 h. After quenching with 10% formic acid, the mixture was transferred in a microspin filter (3 kDa) and centrifuged at ~14,000 *g* for 20 min (4°C). The filtrate was submitted to lyophilization (n.b., the original protocol does not use deferoxamine, degased solutions and argon atmosphere, but we found necessary to be added according to the recoveries studies we performed).

Protocol B (Jaruga et al., 2004): 50 μg DNA were dissolved in 50 μL of 10 mM Tris-HCl buffer pH 7.5 containing 2.5 μL of 1 M AcONa and 45 mM ZnCl_2_ (final pH 6.0). Next, 5 U of Nuclease P1 (in 30 mM AcONa pH 5.3, 5 mM ZnCl_2_ and 50 mM NaCl), 4 mU phosphodiesterase I and 32 U of alkaline phosphatase were added and the mixture was incubated at 37°C. After 24 h, the mixture was transferred in a microspin filter (3 kDa) and centrifuged at ~14,000 *g* for 20 min (4°C). The filtrate was lyophilized overnight.

Protocol C (Belmadoui et al., 2010): 50 μg DNA were dissolved in 100 μL of buffer containing succinic acid 20 mM pH 6, 10 mM CaCl_2_, 3 mM deferoxamine mesylate, and 1 mM EHNA. Next, 5 U of Nuclease P1 (in 30 mM AcONa pH 5.3, 5 mM ZnCl_2_ and 50 mM NaCl), 4 mU phosphodiesterase II and 0.25 U DNAse II were added and the mixture was incubated at 37°C. After 2 h, 10 μL of Tris-HCl buffer 0.5 M pH 8 were added along with 5 U of alkaline phosphatase and 3 mU of phosphodiesterase I. The mixture was incubated for extra 2 h at 37°C. Finally, 10 μL of buffer containing 0.2 M succinic acid pH 6, 0.1 M CaCl_2_ together with 5 U of nuclease P1, 4 mU phosphodiesterase II and 0.25 U DNAse II were added and the mixture was incubated at 37°C for 2 h before being transferred in a microspin filter (3 kDa) and centrifuged ~14,000 *g* for 20 min (4°C). The filtrate was submitted to lyophilization.

Protocol D: 50 μg DNA were dissolved in 100 μL of Ar flushed 10 mM Tris-HCl buffer pH 7.9 containing 10 mM MgCl_2_, 50 mM NaCl, 0.2 mM pentostatin, 5 μM BHT, and 3 mM deferoxamine. Next, 3 U of benzonase (in 20 mM Tris HCl pH 8.0, 2 mM MgCl_2_, and 20 mM NaCl), 4 mU phosphodiesterase I, 3 U DNAse I, 2 mU of PDE II and 2 U of alkaline phosphatase were added and the mixture was incubated at 37°C. Finally the mixture was quenched with 1% formic acid, transferred in a microspin filter (3 kDa) and centrifuged at ~14,000 *g* for 20 min (4°C). The filtrate was submitted to lyophilization.

Protocol E: The same procedure as described in protocol D was followed but after the first incubation, 35 μL of Ar flushed buffer containing 0.3 M AcONa pH 5.6 and 10 mM ZnCl_2_ along with 0.5 U of Nuclease P1 (in 30 mM AcONa pH 5.3, 5 mM ZnCl_2_, and 50 mM NaCl), 4 mU phosphodiesterase II and 125 mU of DNAse II were added. The mixture was incubated at 37°C for extra 21 h, then 20 μL of 0.5 M Tris HCl pH 8.9 were added and the incubation continued for other 2 h, before quenching and removal of the enzymes as described in the previous protocols.

In the cases, when the samples were used for the LC-MS/MS analysis, internal standards of all analytes were added prior to enzymatic digestion.

## Results and discussion

### Quantification of lesions by isotope dilution LC-MS/MS

**Figure 2:**
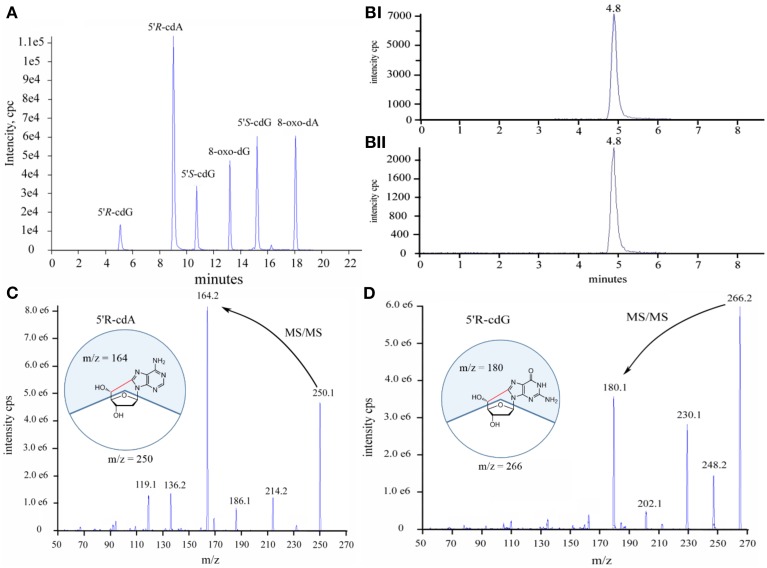
**(A)** Total ion current traces of the six lesions, **(B)** Selective ion current traces of (i) the unlabeled and (ii) isotopic labeled 5′*R*-cdG, **(C)** Diagnostic MS/MS fragmentation profiles of the 5′*R*-cdA, **(D)** Diagnostic MS/MS fragmentation profiles of the 5′*R*-cdG.

Initially we have developed an HPLC-UV analytical protocol for the satisfactory separation of the four natural 2′-deoxyribonucleosides, the four diastereoisomers of the purine 5′,8-cyclo-2′-deoxynucleosides and the two 8-oxo- products of dG and dA (see Figure S11 in Supplementary Material). The quantification of the lesions was based on the liquid chromatography isotope dilution tandem mass spectroscopy technique. The LOD (s/n = 3) and LOQ (s/n = 10) for the 5′*R*-cdG, 5′*S*-cdG, 5′*R*-cdA, 5′*S*-cdA, 8-oxo-dG and 8-oxo-dA were found 0.9, 0.6, 0.6, 0.2, 1.2, 0.2 fmol and 3.1, 2.2, 2.0, 0.8, 4.1 and 0.8 fmol respectively. Noteworthy, the LOD and LOQ values found here are lower than those reported in the literature (Wang et al., 2011, 2012) for the quantification of the lesions in cellular DNA. A typical LC-MS/MS trace (TIC) of the six lesions is depicted in Figure 2A together with the analytical traces of the 5′*R*-cdG and isotopic labeled 5′*R*-cdG (Figure 2B) and the fragmentation profiles of 5′*R*-cdA and 5′*R*-cdG (Figures 2C,D).

**Figure 3:**
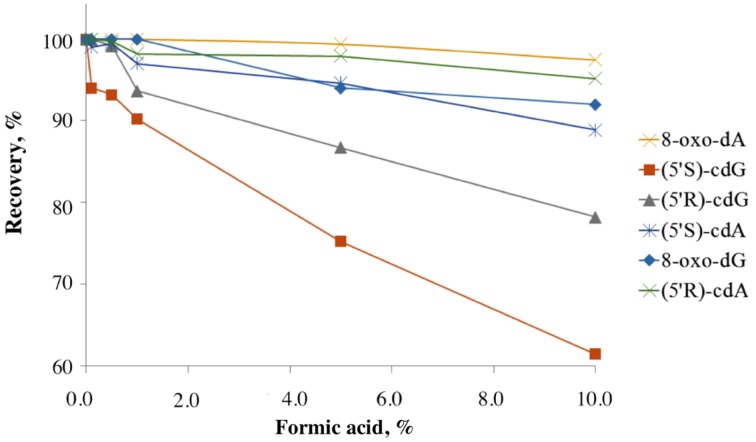
**Recovery of the lesions treated with different percentages of formic acid after incubation at 37°C for 4 h**. The experiments were conducted in triplicates and errors are within the size of the symbols.

Regarding the stability of the lesions under acidic conditions, it is known that the 5′*S*-diastereoisomers of the cdA and cdG are more resistant to acidic hydrolysis than the normal purine 2′-deoxyribonucleosides (dA, dG), the most resistant one being 8-oxo-dG. To our knowledge there is no information regarding the stability of the 5′*R*-diastereomers under acidic hydrolysis conditions (Das et al., 2012). In order to investigate the relative stability of the lesions and the recovery yields under acidic conditions, solutions containing the six lesions were added with different percentages of formic acid and incubated at 37°C for 4 h. The LC-MS/MS analyses showed that all the lesions are highly resistant to acidic hydrolysis and the order of resistance increase following the pattern: 5′*S*-cdG < 5′*R*-cdG < 5′*S*-cdA < 5′*R*-cdA ≈ 8-oxo-dG ≈ 8-oxo-dA. The recoveries of the lesions after 4 h incubation with formic acid are shown in Figure 3.

The investigation of the lesion stability in the enzymatic digestion buffers (pH = 5.6 and pH = 9) and the conditions followed until the LC-MS/MS analysis, autosampler storage, etc., showed that 8-oxo-dG is the only nucleoside resulting in low recoveries when protection from oxidation (use of metal chelators, argon, and antioxidants) is not taken into account. The lesions stock solutions were prepared by taking in to account their extinction coefficients, and particularly for 5′*R*- or 5′*S*-cdG found λ_max_ = 258 nm, ε = 13850 M^−1^ cm^−1^ and for 5′*R*- or 5′*S*-cdA found λ_max_ = 266 nm, ε = 14900 M^−1^ cm^−1^. Regarding the [^15^*N*_5_]-7,8-dihydro-8-oxo-2′-deoxynucleosides were used the values reported in the literature (Taghizadeh et al., 2008).

### Enzymatic digestibility studies

**Figure 4:**
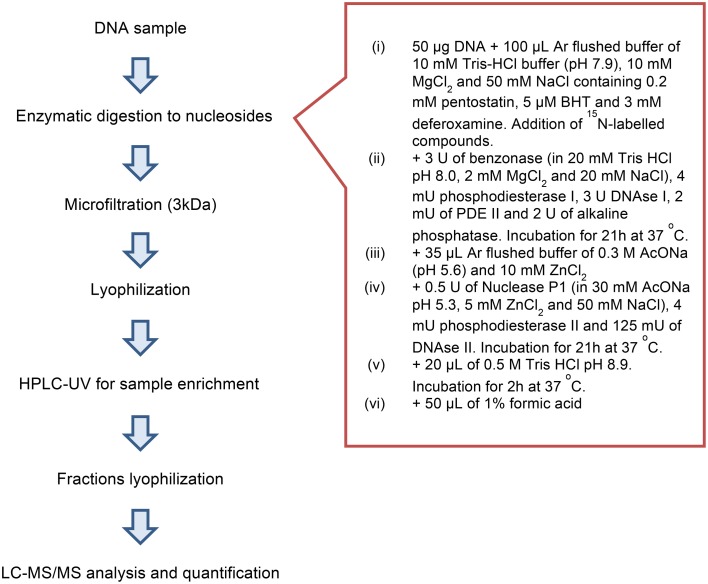
**Flow diagram showing protocol steps**.

**Figure 5:**
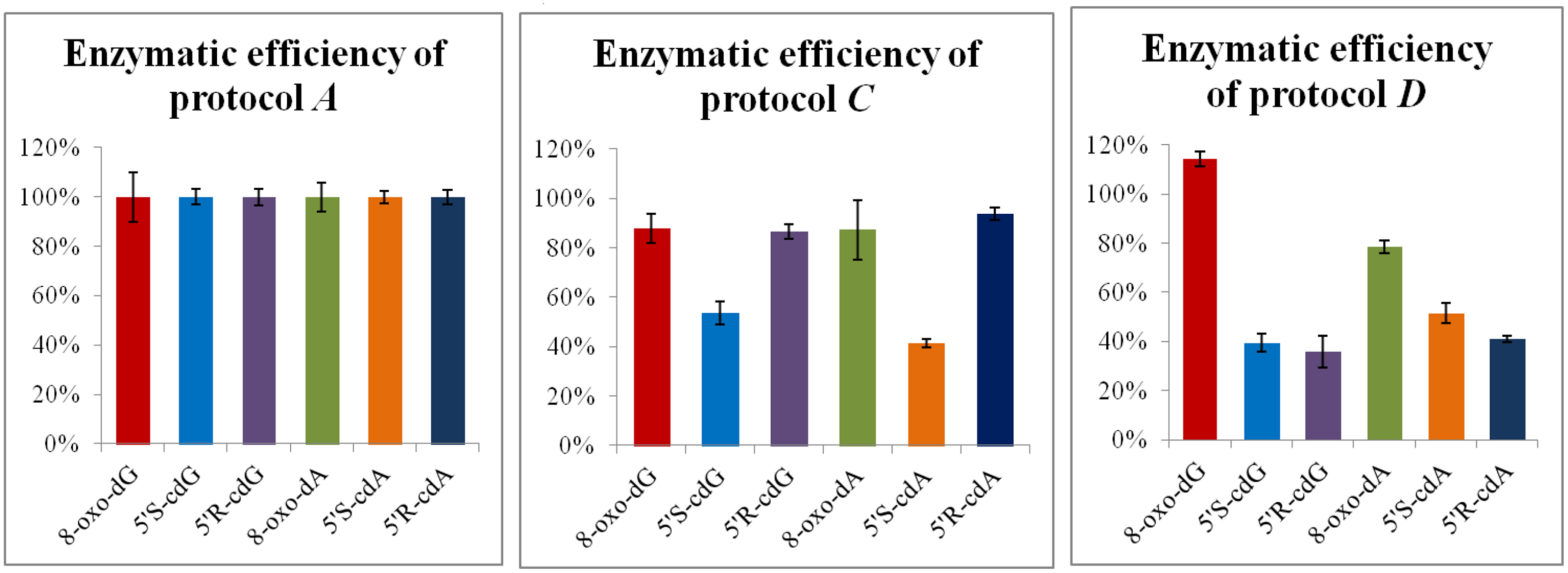
**Digestion efficiency of protocols A, C, and D based on the LC-MS/MS analysis**. The values represent the mean of *n* = 3 independent experiments. The values recorded using Protocol A was employed as 100%.

One of the most important steps for the accurate quantification of the purine 5′,8-cyclonucleosides is the enzymatic digestion for the liberation of the lesions and the natural nucleotides to single molecules. To our knowledge in the literature three relatively recent enzymatic digestion protocols exist (protocols A, B, and C), that are used to release the purine 5′,8-cyclonucleosides from DNA. The amount of the purine 5′,8-cyclonucleosides per million of normal nucleosides (Lesions/10^6^ Nu) produced after gamma irradiation of calf thymus DNA, and the ratio of the 5′*R*/5′*S* diastereoisomers display very different values among the research groups working in this field (Chatgilialoglu et al., 2011). In order to investigate the efficiency of the various enzymatic protocols, we compared them by using the same solution of ds-DNA that was previously split into different aliquots. After incubation the digestion mixtures were filtered by ultracentrifuge filter (3 kDa), the filtrates were lyophilized and resolubilized in ddH_2_O before being injected into the HPLC-UV system. From the analysis it was found that the efficiency of the enzymatic protocols in releasing the single nucleosides varies among protocols. Protocols A and C exhibited similar efficiencies while protocol B reached only 50%. In our two-steps development of the enzymatic digestion protocol we first sough to find which protocol lead to the complete liberation of the unmodified nucleosides. Protocols A and C were found to give the same result (Figure S12) for the digestion of the same DNA samples, while Protocol B reached only up to ~50% in respect to A or C. When other protocols liberate the same amount of unmodified nucleosides, then they can be further considered also for their efficiency in liberation of the other modified nucleosides (i.e., cyclonucleosides). In fact, we evaluated the protocols A and C based on their ability to liberate also the lesions. This evaluation showed that Protocol A was predominant over Protocol C (**Figure 5**). The new digestion protocol was designed based on the enzymatic combination of benzonase and P1 nucleases. Benzonase was successfully used in the quantification of 5-met-Cyt and 8-oxo-dG (Quinlivan and Gregory, 2008; Cui et al., 2013), however the choice of the other enzyme to couple and the exact steps and duration of the treatment required to be determined. The optimization of the enzymatic protocol was performed in two phases. Initially, ds-DNA was incubated with different amount of benzonase, DNAse I, alkaline phosphatase, and phoshodiesterase I in 20 mM Tris-HCl buffer at pH 8 containing 20 mM MgCl_2_. It was found that the presence of 20 mM NaCl increases the reactivity of the enzymes and the HPLC-UV analysis showed that the digestion was complete after 21 h incubation at 37°C with 2 U of benzonase, 2 U DNAse, 2 U alkaline phosphatase, and 2 mU phoshodiesterase I. Figure 4 summarize the protocol steps in a flow diagram (see also Figure S12 in Supplementary Material for the relative efficiency of the four protocols).

Next, DNA solution (0.5 mg/mL) flushed with Ar, irradiated in a Gammacell ^60^Co with 60 Gy (4.1 Gy/min) of irradiation dose at ambient temperature, was split in different aliquots of 50 μL each, and incubated according to the described protocol D. For comparison, some of the ds-DNA aliquots were digested according to Protocols A and C. Identical analytical results were achieved for the normal nucleosides, as quantified after the HPLC-UV analysis. On the other hand, the LC-MS/MS quantification of the lesions in the above samples revealed that the protocol D is much less efficient on the liberation of the purine 5′,8-cyclo-2′-deoxynucleosides compared to protocol A, even after increment of the enzyme units and the incubation times (Figure 5). Considering the results for 8-oxo-dG, any of the protocols A, C, or D gave the same value.

Stability studies of the lesions in the buffer used during the enzymatic digestion with protocol A showed that special precautions are necessary for keeping the recovery close to 90% (ESCODD, 2002). Particularly, the combination of deferoxamine, BHT, inert atmosphere, and argon-flushed buffers is used as described elsewhere (Taghizadeh et al., 2008; Thornalley et al., 2010). Interestingly, the control samples of the calf thymus DNA, that were not exposed to γ-rays, were found to contain all four cyclopurines and the two 8-oxo lesions (see Table S4 in Supplementary Material) (Cui et al., 2013; Guerrero et al., 2013).

**Figure 6:**
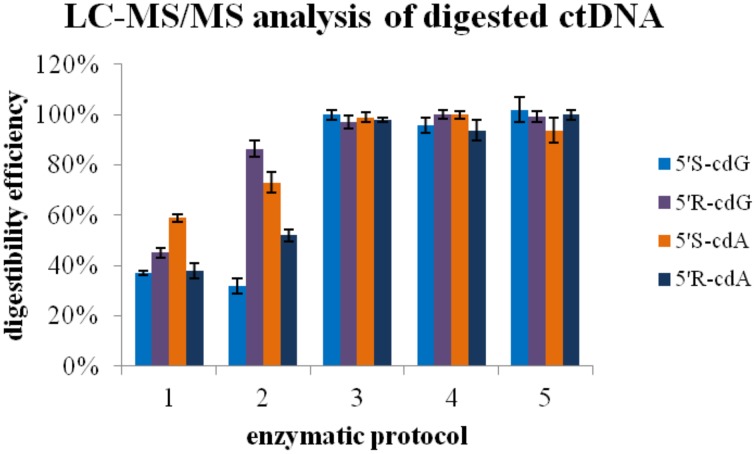
**Enzymatic protocol vs. digestibility efficiency 1 Application of Protocol D 2 Protocol D and then second digestion with 20 mU PDE II (pH 5.6) 3 Protocol D and then second digestion with 1U nuclease P1 and 20 mU PDE II (pH 5.6) 4 Protocol D and then second digestion with 1U nuclease P1 5 Protocol D, then 2nd digestion with 1U nuclease P1 (pH 5.6) and final third digestion with alkaline phosphatase and PDE I (pH 8.9)**. The values recorded using Protocol A was employed as 100%.

In order to identify the enzyme that can cleave the phosphodiester bond in the remaining DNA fragments, after treatment according to protocol D (Figure 6; ep1), the digestion mixture was split to different aliquots and a buffer of 300 mM AcONa pH 5.3 containing 10 mM ZnCl_2_ was added (until final pH 5.8) together with 20 mU phosphodiesterase II (Figure 6; ep2). The same procedure was followed also with a combination of 20 mU phosphodiesterase II and 1 U nuclease P1 (Figure 6; ep3), and by using only 1 U of nuclease P1 (Figure 6; ep4). Finally, aliquots were treated with a combination of phosphodiesterase II, 1 U nuclease P1, DNase II according to protocol E (Figure 6; ep5).

Studies on the analytical recoveries were conducted by splitting a calf thymus DNA solution in multiple aliquots, spiking half of them with the lesions before being digested according to the enzymatic protocol E, and comparing the added amount with calculated by the LC-MS/MS system, after subtraction of the pre-existed in DNA amount of lesions. The calculated recoveries for 5′*R*-cdG, 5′*S*-cdG, 5′*R*-cdA, 5′*R*-cdA, 8-oxo-dG, and 8-oxo-dA were found 104, 95, 96, 90, 90, 113% respectively and the coefficients of variation as 2–5%. No significant loss was recorded during the sample workup, storage, and until the LC-MS/MS analysis.

In this work all the published digestion protocols have been applied to the same aliquots containing DNA irradiated with 60 Gy irradiation dose (γ-rays). Using this sampling, it was found surprisingly that the efficiency of the digestion protocols for the release of the single unmodified nucleosides is dissimilar in the case of Protocol B (Figure S12 in Supplementary Material). Additionally, when nuclease P1 was replaced by benzonase (Protocol D) the efficiency of the lesion release could be calculated as 30–60% (Figure 6). In order to identify the enzyme that can catalyze the hydrolysis of remaining DNA fragments containing the lesions, nuclease P1, and phosphodiesterase II were used either separately or together in the first digestion aliquots. In agreement with previous studies (Romieu et al., 1998) the LC-MS/MS analysis revealed that only nuclease P1 is able to lead to a complete digestion. Moreover, a subsequent activation of alkaline phosphatase by changing pH to basic values was found not to be necessary. The distortion that the purine 5′,8-cyclo-2′-deoxynucleosides cause to the active sites of phoshodiesterases can likely inhibit the formation of the necessary interactions for the hydrolysis of nucleotidyl-enzyme intermediate by the threonine active-site. On the contrary, the zinc-finger in the active site of nuclease P1, activated by a water molecule, was found to promote the hydrolysis at long incubation times. Noteworthly, nuclease P1 is known to hydrolyse native DNA at much lower rate than the heat-denaturated DNA (Fujimoto et al., 1974) and it is much more expensive than benzonase, which is also more stable and known to hydrolyse fast both single and double stranded DNA. The nuclease P1 buffer used, in terms of zinc cation concentration, remained unaltered in respect to the ones reported elsewhere (Romieu et al., 1998; Wang et al., 2011), since our new protocol employs an off-line HPLC clean-up that eliminates the problems related to the mass detector deriving from the presence of non-volatile ions in the samples. In agreement to our results, recently it has been reported that the bulky spiroiminodihydantoin 2′-deoxynucleoside lesions challenge the hydrolytic activity of the 3′-exo and 5′-exo phosphodiesterases, and nuclease P1 quantitatively hydrolyzes the phosphodiester bond between the modified (lesion) and the normal nucleoside (Chen et al., 2013).

## Conclusions

The use of purine 5′,8-cyclonucleosides as marker of DNA damage and reporter of DNA molecular asset at the moment of the HO^**·**^ insult is increasingly appreciated in free radical research, together with the advantage that cyclopurine markers do not suffer the stability problems and artifactual oxidative process of the most known 8-oxo-dG. In this paper we applied a step-wise approach for the development, optimization and validation of a new analytical procedure allowing the reproducible and quantitative determination of cdA, cdG and the two 8-oxo-lesions. Under the actual form, the procedure offers important ameliorations to apply extensively the protocol to the affirmation of cyclopurine biomarkers in different health conditions. The role of the zinc finger nuclease P1 as the only suitable enzyme, from those tested, for the quantitative liberation of the single nucleoside lesions was assessed. Moreover, we reduced by ~10 fold the cost of the analysis per sample with the introduction of the nuclease benzonase.

### Conflict of interest statement

The authors declare that the research was conducted in the absence of any commercial or financial relationships that could be construed as a potential conflict of interest.
