# Supplementary material for: An ameliorative protocol for the quantification of purine 5′,8-cyclo-2′-deoxynucleosides in oxidized DNA
Source: Front Chem. 2015 Jul 28;3:47. doi: 10.3389/fchem.2015.00047 (PMC4517065; doi:10.3389/fchem.2015.00047)
Supplement: Supplementary file 1 [file DataSheet1.PDF]

Supplementary Material

An ameliorative protocol for the quantification of purine 5',8-cyclo-2'-deoxynucleosides in oxidized DNA

Michael A. Terzidis[1] and Chryssostomos Chatgilialoglu[1,2*]

[1] Istituto per la Sintesi Organica e la Fotoreattività, Consiglio Nazionale delle Ricerche, Via P. Gobetti 101, 40129 Bologna, Italy
[2] Institute of Nanoscience and Nanotechnology, N.C.S.R. "Demokritos", 15310 Agia Paraskevi, Athens, Greece

*** Correspondence:** Chryssostomos Chatgilialoglu, Institute of Nanoscience and Nanotechnology, N.C.S.R. "Demokritos", 15310 Agia Paraskevi, Athens, Greece
c.chatgilialoglu@inn.demokritos.gr or chrys@isof.cnr.it

Synthesis of reference compounds and internal standards

HPLC Purification of 5'*R*- and 5'*S*-cdA after photolysis of 8-Br-dA

HPLC purification (Agilent 1100 series) on C18 reverse phase column [Phenomenex, Luna 5µm, C18 (2), 100Å, 4.6 mm ×150 mm] from the photo-irradiation of 1mM 8-bromo-2'-deoxyadenosine in acetonitrile. The flow was regulated to 1 mL/min and the gradient was 2mM ammonium formate (A) and acetonitrile (B), B 0% → 0.3% from 0 → 2.2 min, 0.3% → 0.8% from 2.2 → 6.2 min, 0.8% → 1% from 6.2 → 11 min, 1% → 1% from 11 → 20 min, 1% → 8% from 20 → 26 min, 8% → 10% from 26 → 30 min, 10% → 30% from 30 → 35 min. The fractions, from 16 → 18 min and from 27 → 28.2 min, containing the peaks of the corresponding products (Figure S1)

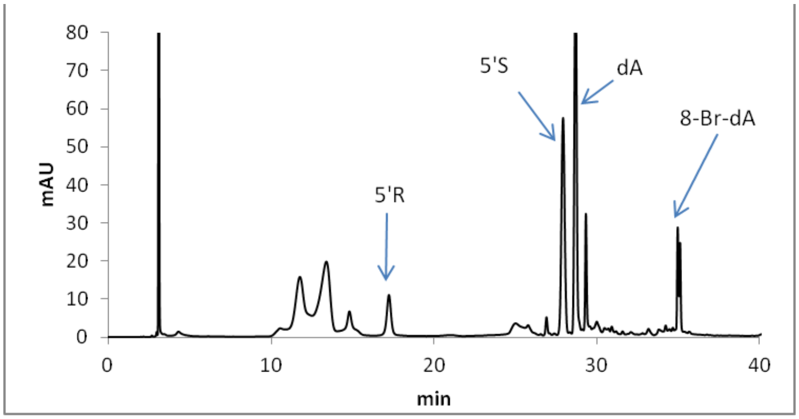

FIGURE S1. Chromatogram of 1 mM 8-Br-dA acetonitrile solution after 15 min with UV-vis light (125 W medium pressure Hg lamp) under oxygen free conditions.

Synthesis of ^{15}N labeled derivatives

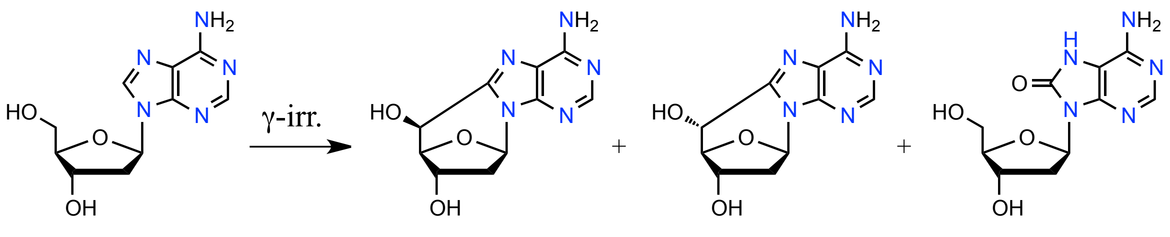

[$^{15}N_5$]-dA

FIGURE S2. The synthesis of adenine derivatives was performed as previously reported for the unlabelled compounds. In particular, 1 mL of aqueous solution containing 1.5 mM of [$^{15}N_5$]-dA was prepared, flushed with N_2O for 15 min and irradiated with a total dose of 2 kGy at a dose rate of 4.5 Gy min^{-1}. The crude mixture was submitted to HPLC purification (see Fig. S3).

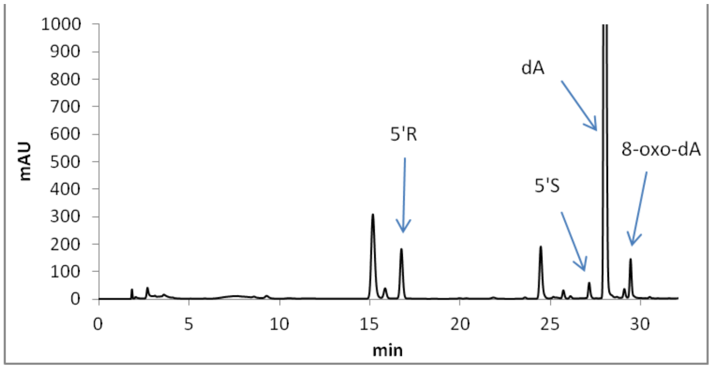

FIGURE S3. Chromatogram of N_2O saturated 1 mM [$^{15}N_5$]- 2'-deoxyadenosine aqueous solution after 2 kGy of gamma radiolysis.

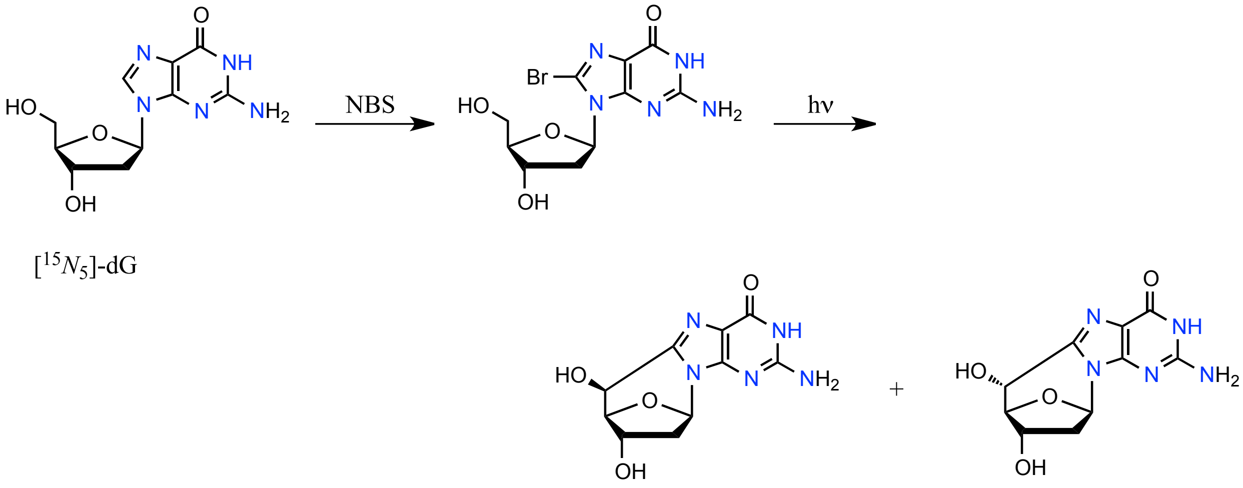

FIGURE S4. The synthesis of [$^{15}N_5$]-8-bromo-2'-deoxyguanosine has been achieved following a known procedure for the unlabelled compound. Particularly, 1 mg (3.5 μmol) of [$^{15}N_5$]-dG was suspended to 500 μL of acetonitrile/water mixture 4:1 in a microcentrifuge tube of 1.5 mL volume. Then 1.3 mg N-bromosuccinimide (7.3 μmol) was added to the suspension in three portions and the mixture stirred at room temperature for 2 h. After the solvent was removed under a stream of argon, acetone (100 μL) was added. The mixture was stirred at room temperature for 4 h and then stored at -20 °C overnight. Next, the mixture was centrifuged at 1000g for 1 min and the supernatant was removed with a pipette. The precipitate was washed with cold acetone (60 μL), centrifuged again and the liquid removed with a pipette. The solid [$^{15}N_5$]-8-bromo-2'-deoxyguanosine was dried under vacuum, weighted and 1 mM solution in ddH_2O was prepared. The solution was then transferred into the UV irradiation apparatus, flushed with argon for 15 min and irradiated for 30 min with a UV light (5W low pressure Hg lamp). The reaction mixture was quenched with a 5% $NaHCO_3$ solution (final pH 7) and submitted to HPLC for purification (see Fig. S5).

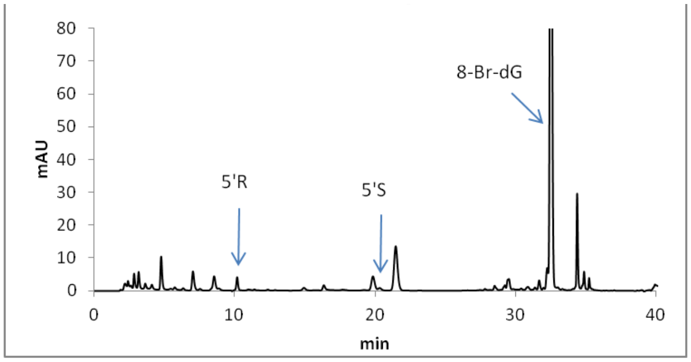

FIGURE S5. Chromatogram of 1 mM [$^{15}N_5$]-8-bromo-2'-deoxyguanosine aqueous solution after 30 min photolysis with UV light under oxygen free conditions.

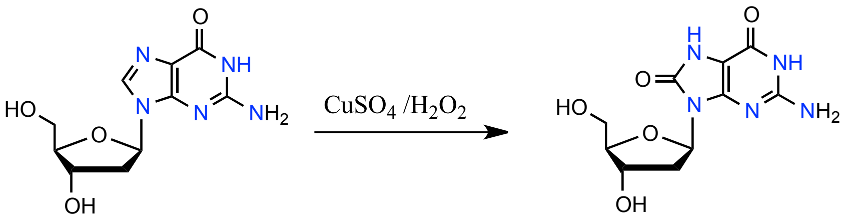

[$^{15}N_5$]-dG

FIGURE S6. [$^{15}N_5$]-dG (0.24 mg, 0.82 μmol) was dissolved in 164 μL of water in an ultrasound bath. After, 5.45 μL of 0.5 M freshly prepared ascorbic acid (2.7 μmol) were added followed by 3.3 μL of 0.1 M $CuSO_4$ (3.3 μmol) and 9.4 μL of 30% hydrogen peroxide. The reaction mixture was stirred at room temperature and quenched after 2h with a 5% Na_2SO_3 solution. The crude mixture was submitted to HPLC purification (see Fig. S7).

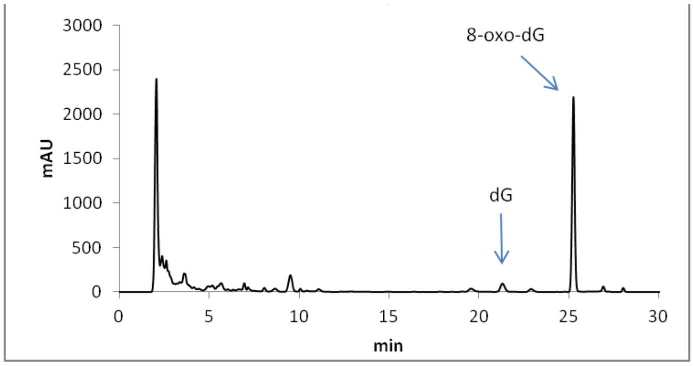

FIGURE S7. Chromatogram of aqueous [$^{15}N_5$]-dG solution after the reaction with $CuSO_4$ /H_2O_2 in presence of ascorbic acid.

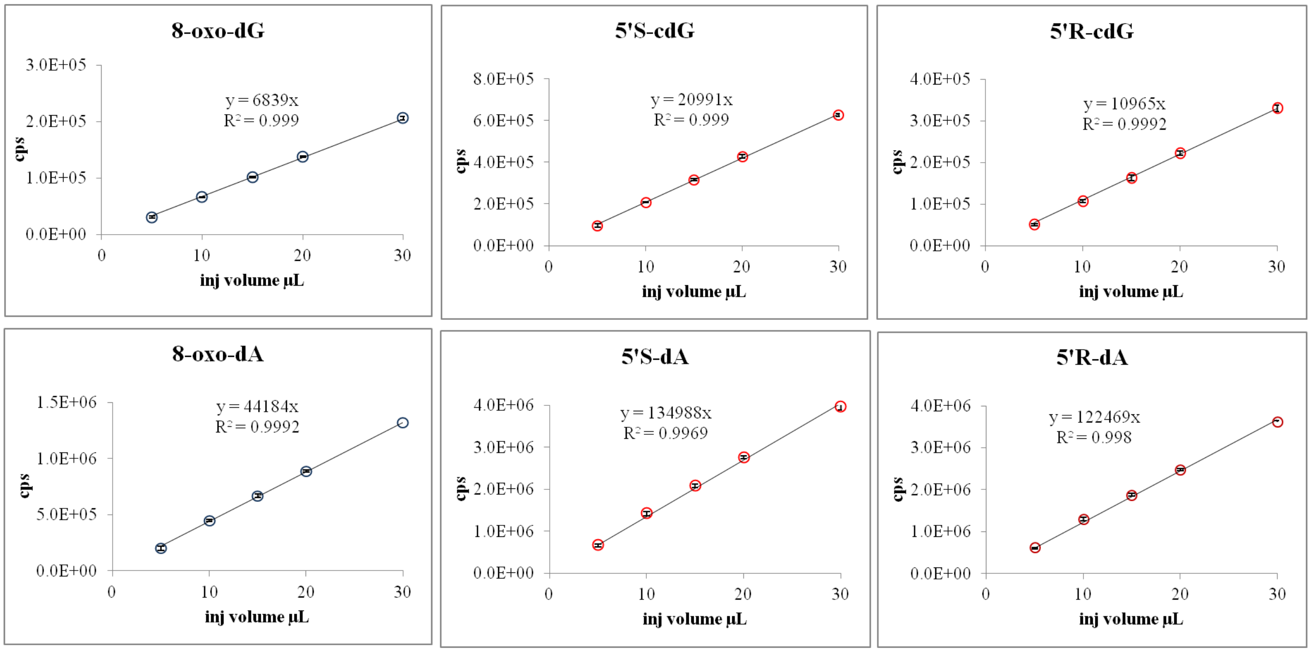

FIGURE S8. The MRM signal areas of the lesions (10 ng/mL standards solution) against different injection volumes. Each data point is the mean of $n = 3$ independent measurements and the uncertainties are the standard errors.

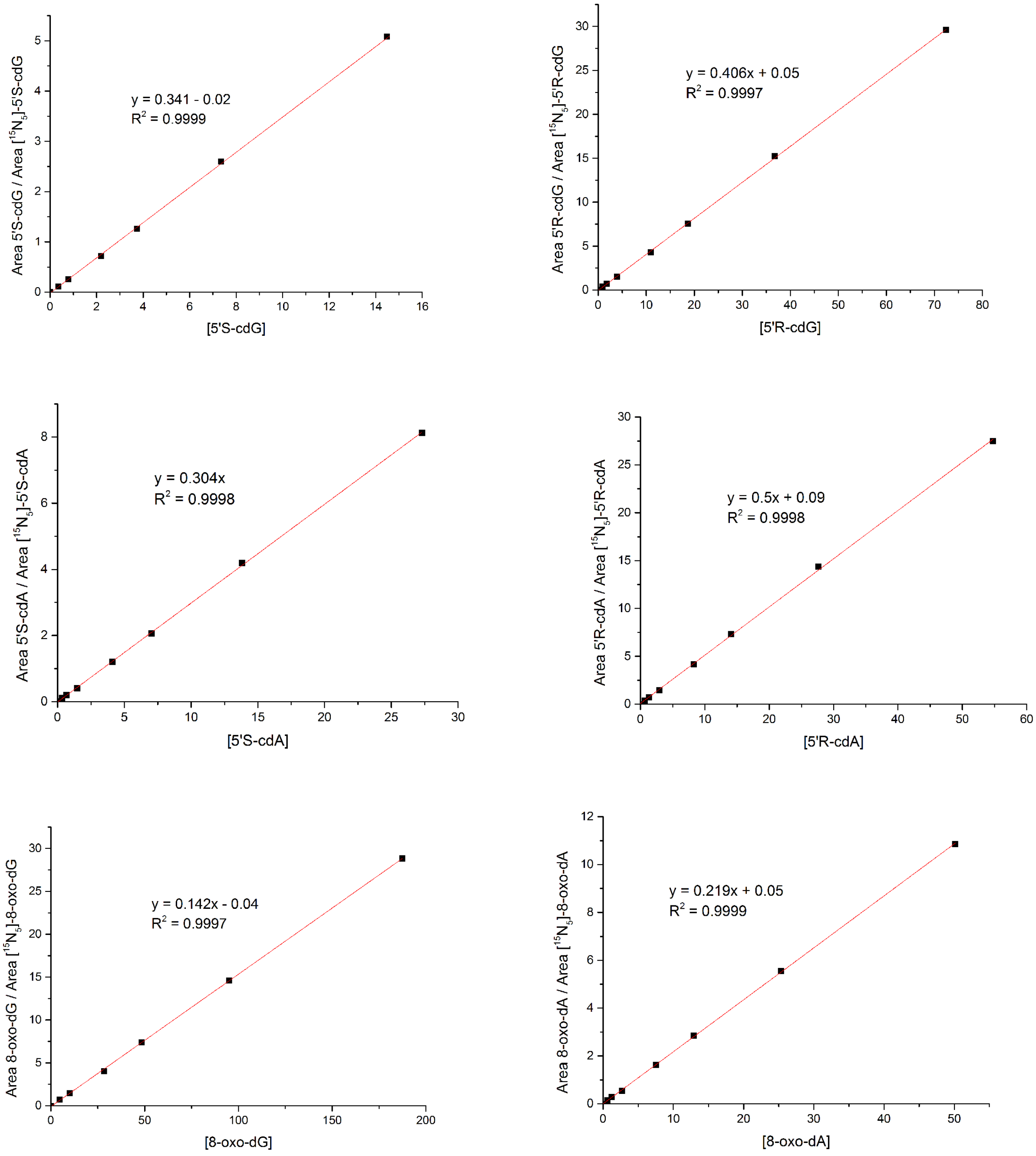

FIGURE S9. Calibration curve for the quantification of six lesions (nM)

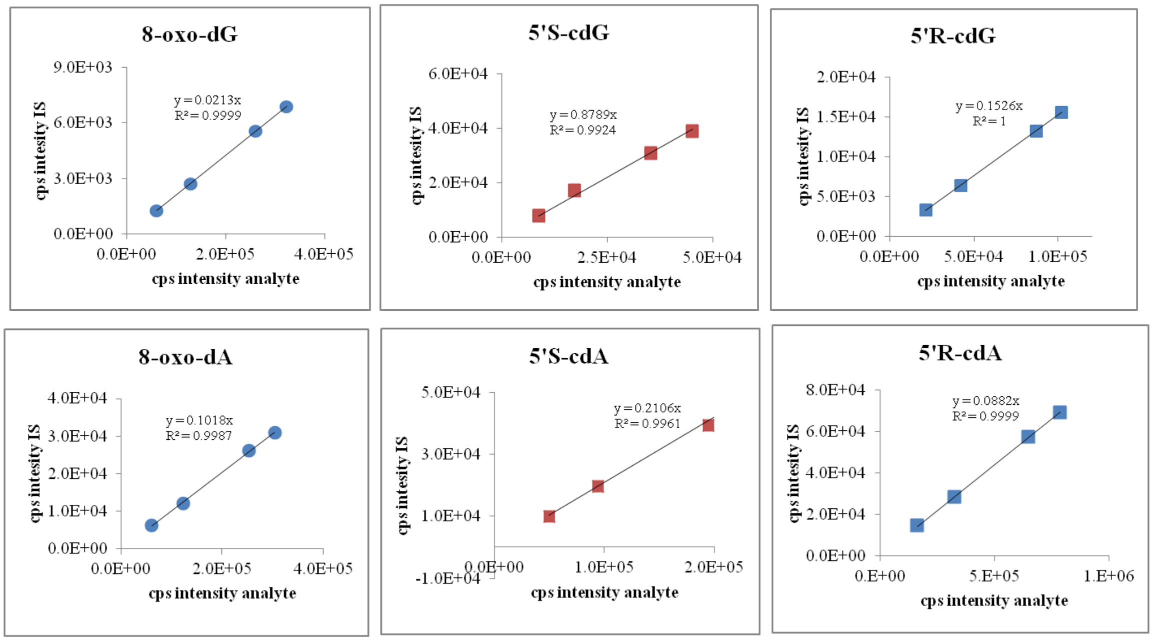

FIGURE S10. The MRM signal areas of the internal standards (IS) against the MRM signal areas of the lesions upon dilution (dilution factors 0, 1.25, 2.5, 5).

Determination of the lesions tandem mass CID product ion spectra

For transferring the literature reported calibration parameters (Belmadoui et al., 2010), the triple quadrupole tandem mass spectrometer (ABSciex 4000 ion-Trap, ABSciex Inc.,Canada) was tuned by using 100% aqueous standard solutions of the 5'*R*-cdG and 5'*S*-cdG and 95:5 water / acetonitrile solutions for the rest of the lesions (1 μg/ml) . The analytes were introduced to the Turbo-V source by continuous infusion with a Harvard model 22 syringe pump (Harvard Apparatus Ltd., Edenbridge, UK). The flow rate of the pump was chosen carefully to give intensities between 10^5 and 10^6 counts per second (cps) for each analyte. The TurboIonSpray® probe operated in positive ion mode with an ion spray voltage of 5.5 kV (IS) and nitrogen as collision gas (CAD) at medium. The probe temperature, curtain gas and nebulizing gas 1 and 2 were set to 650 °C, 15 psi, 80 psi and 60 psi, respectively. The schedule MRM optimized parameters were the declustering potential (DP), the entrance potential (EP), the collision energy (CE), and the cell exit potential (CXP) and are reported in **Table S1**. The detection window set to 180 sec with target scan time of 1 sec. The pause between the mass rages was set to 5 ms. Unit mass resolution was set for both the Q1 and Q3 quadrupole. Data were acquired and processed using Analyst 1.5.2™ software (AB Sciex).

Quantification of the purine 5',8-cyclonucleosides by isotope dilution LC-MS/MS

The quantification of the lesions was based on liquid chromatography isotope dilution tandem mass spectroscopy technique, as described above. Trials of analytical methods starting with 0% of organic solvent showed instability of the retention time of the first eluted analyte (5'*R*-cdG). However starting with at least 1% of acetonitrile gave stable and reproducible retention times and also improved the shape of the peaks. The LC-MS/MS method produced linear response to the lesions and was found highly sensitive with low variability. The parameters used for the calibration of the mass detector are reported in **Table S1**.

Table S1. Parameters used for the detection of the purine 5',8-cyclonucleosides and 8-oxo oxidation products by isotope dilution tandem mass spectrometry in multiple reaction monitoring mode.

**Lesion**[1]	$\mathbf{R_t}$[2]	**Transit.**[2]	**Q1**	**Q3**	**Q1**	**Q3**	**DP**	**EP**	**CE**	**CXP**
			Unlabeled		$^{15}N_5$ labeled					
5'*R*-cdG	5.1	Quant.[2]	266.2	180.1	271.2	185.1	60	10	25	10
		Qualit.[2]	266.2	202.1	271.2	206.1	60	10	35	12
5'*R*-cdA	9.0	Quant.	250.1	164.2	255.2	169.2	65	10	20	9
		Qualit.	250.1	136.2	255.2	141.2	65	10	40	6
5'*S*-cdG	10.7	Quant.	266.2	180.1	271.2	185.1	60	10	30	10
		Qualit.	266.2	202.1	271.2	207.1	60	10	35	11
8-oxo-dG	13.2	Quant.	284.1	168.2	289.1	173.2	50	10	20	8
		Qualit.	284.1	140.1	289.1	145.1	50	10	45	7
5'*S*-cdA	15.2	Quant.	250.2	164.1	255.2	169.1	65	10	25	9
		Qualit.	250.2	136.2	255.2	141.2	65	10	40	6
8-oxo-dA	18.1	Quant.	268.1	152.1	273.1	157.1	50	10	20	10
		Qualit.	268.1	125.1	273.1	130.1	50	10	50	5

[1]The analyses performed in positive mode. The mass spectrometer capillary was adjusted to 6 mm, which found to give the best response for the analytes. [2]R_t = retention time in minutes, Transit. = transition mode, Quant. = quantitative, Qualit. = qualitative

Table S2. Equations of the response curves constructed by comparing the MRM signal areas of the lesions and their internal standards.

Analyte	Equation	R^2
8-oxo-dG	y = 0.142x − 0.04	0.9997
5'*R*-cdG	y = 0.406x + 0.05	0.9997
5'*S*-cdG	y = 0.341x − 0.02	0.9999
8-oxo-dA	y = 0.219x + 0.05	0.9999
5'*R*-cdA	y = 0.500x + 0.09	0.9998
5'*S*-cdA	y = 0.304x	0.9998

Where y stands for [(Area of Analyte) / (Area of Analyte Internal Standard)] and x for (nM Concentration of the Analyte). The amount of the labeled compounds used for the contraction of the calibration curves and the lesions quantifications were kept always constant (see Table S3).

Table S3. Concentrations (nM) of the lesions used for the construction of the calibration curves.

5'*R*-cdG	5'*S*-cdG	5'*R*-cdA	5'*S*-cdA	8-oxo-dG	8-oxo-dA
73	15	56	28	190	50
37	7.5	28	14	97	26
22	4.4	17	8.3	57	15
7.9	1.6	6.0	3.0	20	5.4
3.6	0.7	2.7	1.4	9.3	2.5
1.7	0.3	1.3	0.7	4.4	1.2
0.0	0.0	0.0	0.0	0.0	0.0

All the solutions prepared for the calibration curves and all the samples were spiked with $^{15}N_5$ isotopic internal standards mixtures containing: $^{15}N_5$-5'*R*-cdG 15 fmol, $^{15}N_5$-5'*S*-cdG 15 fmol, $^{15}N_5$-5'*R*-cdA 20 fmol, $^{15}N_5$-5'*S*-cdA 20 fmol, $^{15}N_5$-8-oxo-dG 65 fmol, $^{15}N_5$-8-oxo-dA 40 fmol.

Table S4. Levels (lesions/10^6 nucleosides) of cdG, cdA, 8-oxo-dA and 8-oxo-dG in calf thymus DNA not exposed to γ-rays (controls). The data represent the means and standard deviations of n = 8 independent experiments from 2 different DNA batches.

5'*R*-cdG	5'*S*-cdG	5'*R*-cdA	5'*S*-cdA	8-oxo-dG	8-oxo-dA
9.8±2.7	3.2±0.5	7.4±1.8	3.7±0.8	424±135	16.1±5.1

Quantification of the normal nucleosides by HPLC-UV

We developed an analytical protocol for the separation of the four normal 2'-deoxyribonucleosides, the four diastereoisomers of the purine 5',8-cyclo-2'-deoxynucleosides and the two 8-oxo-derivatives of dG and dA, respectively. The optimal HPLC chromatographic separation (monitored at 260 nm) was achieved at 25 °C by using a 4.6 mm x 150 mm Luna C18 (2) 100 Å column (5μ min particle size, Phenomenex) loaded with a pre-column C18 (2) cartridge, on an Agilent 1100 HPLC-UV system (Agilent, US). The gradient used for the separation of the analytes (**Table S5**) involves 2 mM ammonium formate, acetonitrile and methanol as solvents. The involvement of methanol (solvent C) in the washing step of the method was crucial for avoiding carry-over phenomena of the nucleosides during the analytical runs. Calibration curves were prepared and the quantification of the normal nucleosides was based on their absorbance at 260 nm. The variation of the temperature ±1 °C during the runs was found unimportant for the retention times, however variations more than 3 °C influenced the retention times, but not the chromatographic separation. The collection of the lesions was performed at time collection mode and time windows as reported in **Table S6**.

Table S5. The gradient used for the separation of the analytes for the samples clean-up and quantification of the unmodified nucleosides.

Time (min)[1]	0	2.2	6.2	11	12	18	26	30	35	37	39	45
Solvent A[2]%	100	99.7	99.2	98.5	98.1	98.1	92	90	70	60	100	100
Solvent B[3]%	0	0.3	0.8	1.5	1.9	1.9	8	10	30	0	0	0
Solvent C[4] %	0	0	0	0	0	0	0	0	0	40	0	0

[1] The flow rate remained constant at 1 mL/min during the analyses. [2] Solvent A: 2 mM ammonium formate. [3] Solvent B: acetonitrile. [4] Solvent C: methanol.

Table S6. The time windows used for the collection of the lesions during the HPLC clean-up.

Lesion	5'*R*−cdG	5'*R*−cdA	5'*S*−cdG	8−oxo−dG	5'*S*−cdA	8−oxo−dA
Min*	9.5−10.8	16−17.5	19.7−21	24.6−26	26.7−27.7	29−31.0

* Collection time window in minutes. The room temperature were the analysis was performed was at 1 ± 25 °C during the analyses.

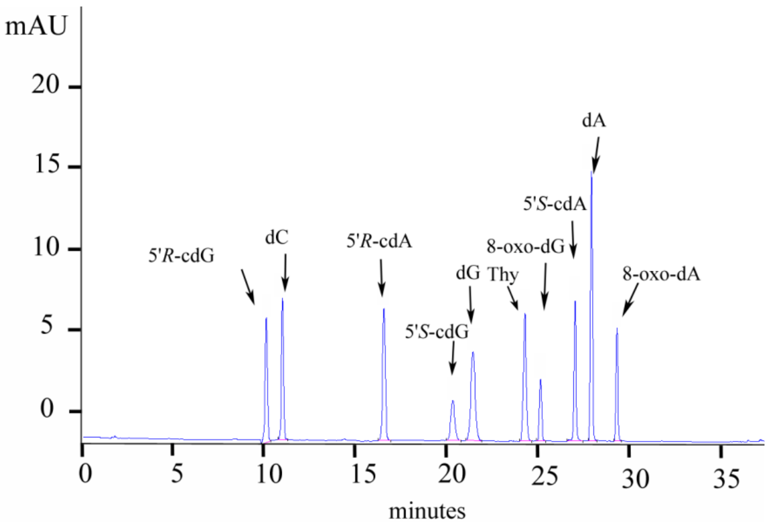

FIGURE S11. Optimal HPLC separation (monitored at 260 nm) of the ten nucleosides.

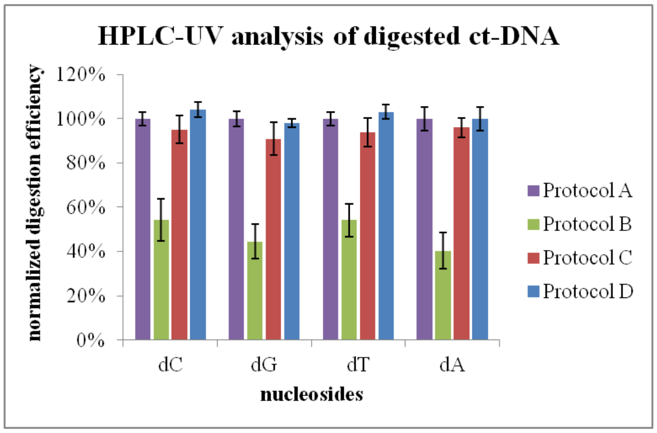

FIGURE S12. Digestion efficiency of protocols A, B, C and D. The values represent the mean of $n = 3$ independent experiments. The values recorded using Protocol A was employed as 100%.
